# Improving Corrosion Resistance of Magnesium Alloy in Cl^−^ Containing Simulated Concrete Pore Solution by Ultrasound-Assisted Chemical Deposition

**DOI:** 10.1155/2021/5462741

**Published:** 2021-07-16

**Authors:** Ye Wang, Guosong Wu

**Affiliations:** College of Mechanics and Materials, Hohai University, Nanjing 211100, China

## Abstract

Coatings are playing an important role in corrosion mitigation of magnesium alloys, and in this study, a facile and eco-friendly chemical deposition process is proposed to improve the corrosion resistance of magnesium-neodymium alloys. The mixture of 1.5 mol/L KH_2_PO_4_ solution and 1.2 mol/L CaCl_2_ solution is used for reaction solution, and ultrasound is introduced into the process for assisting the chemical deposition. After 40 minutes of the surface treatment, the surface and cross-sectional morphologies are observed by scanning electron microscope (SEM), which reveals that a layer of dense coating is formed on Mg alloy. Energy-dispersive X-ray spectroscopy (EDS) and X-ray Diffraction (XRD) are further combined to analyze the coating, and it is thereby confirmed that this coating mainly consists of CaHPO_4_·2H_2_O. Electrochemical tests and soaking experiments are conducted to evaluate the corrosion resistance of the treated samples in simulated concrete pore solutions. Both the untreated and treated samples have a good corrosion resistance in the Cl^−^ free simulated concrete pore solution, but their corrosion behavior is influenced by the introduction of Cl^−^ in this study. Fortunately, the coating can protect the substrate effectively in the Cl^−^ containing simulated concrete pore solution. In summary, it provides a possible way for magnesium alloys to improve their corrosion resistance when they are used in building engineering.

## 1. Introduction

Magnesium alloys have received much attention in the aerospace, automotive, electronic, and biomedical industries due to their low density, high specific strength, and natural biodegradability. However, poor corrosion resistance is one of their fatal disadvantages to hinder their further engineering applications [1–7]. Usually, coating is an effective means to improve the corrosion resistance of Mg alloys in aqueous solutions and many kinds of techniques have ever been attempted [8–11]. Compared to chemical methods, most physical methods such as physical vapor deposition and ion implantation are innocuous to environment [12–14]. But the related equipment is always expensive and not suitable for large-scale engineering. Therefore, it is very imperative to develop new green chemical methods for the surface treatment of magnesium alloys.

Lightweight construction has been gradually recognized in modern civil engineering, and steel is expected to be replaced with lighter structural materials. For example, aluminum alloy bars have been considered an alternative to steel rebars in concrete structures. But, their native oxide coating is not stable in acid (pH <4) or alkaline (pH >9) environments, and aluminum alloy bars are often susceptible to corrosion in the alkaline environment of concrete structures [15]. In fact, magnesium alloys are lighter and have higher specific strength than aluminum alloys. Furthermore, magnesium has good immune behavior in alkaline aqueous environments [16, 17], which may make magnesium alloys adapt the alkaline concrete environment. However, chloride ions may appear in the concrete environment and affect the corrosion behavior of magnesium alloys. Therefore, it is essential to develop a facile and eco-friendly surface treatment process to protect magnesium alloys. Ultrasound has often been introduced into the chemical reaction process and can assist the formation of dense coating without using additional heating [18]. Calcium-phosphorus (Ca-P) coatings have been paid more attentions due to their excellent corrosion resistance [19]. For instance, Li et al. have applied the hydrothermal method to fabricate a glucose-induced phosphate coating on pure magnesium [20]. But most of the processes are complicated and time-consuming, which are not suitable for civil engineering. In this study, a calcium-phosphorus coating is prepared on the surface of Mg-3.3 wt. %Nd alloy by ultrasound-assisted chemical deposition, and its corrosion behavior is also investigated in simulated concrete pore solutions.

## 2. Materials and Methods

As-cast Mg-3.3 wt. %Nd alloys were used for substrate materials and cut into 10 mm × 10 mm × 5 mm pieces in this investigation. First, the samples were mechanically ground with SiC paper up to # 1200 and then polished with Al_2_O_3_ pastes. Second, they were rinsed with ethanol at room temperature and rapidly dried with hot air. Third, 1.5 mol/L KH_2_PO_4_ and 1.2 mol/L CaCl_2_ were prepared, respectively. Here, a conventional ultrasonic cleaner was used for producing ultrasound, and a beaker with 30 mL of the mixture was placed into the ultrasonic cleaner. After the Mg alloy samples were treated in the beaker at a frequency of 40 kHz for 40 minutes, they were taken out, washed with deionized water and ethanol in turns, and then naturally dried in air at room temperature.

Field emission scanning electron microscope (FESEM) was performed to observe the surface and cross-section morphology of the treated sample, and the elemental distribution of the treated one was analyzed by energy-dispersive X-ray spectrometer (EDS). Grazing incidence X-ray diffraction (GIXRD) was performed to characterize the surface phase composition of the treated sample, and two different angles of X-ray incidence *α* (*α* = 1° and 5°) were selected in this investigation.

In order to simulate the alkaline environment in the concrete construction, a saturated Ca (OH)_2_ solution was used as the simulated concrete pore solution. In addition, the saturated Ca (OH)_2_ solution was further diluted by 3.5 wt.% NaCl solution with a volume ratio of 1 : 1 to evaluate the effect of chloride ions. Electrochemical test and soaking experiment were applied to evaluate the corrosion behavior in the above solutions. Electrochemical corrosion tests were conducted on a CHI660E electrochemical workstation using the conventional three-electrode technique. Here, the potential was referenced to a saturated calomel electrode (SCE), and the counter electrode was a platinum sheet. The specimen with an exposed surface area of 1 × 1 cm^2^ was immersed in 200 mL simulated concrete pore solution. After immersion for 30 min, the electrochemical impedance spectra (EIS) were collected from 100 kHz to 100 mHz with a 5 mV sinusoidal perturbing signal at the open-circuit potential. After the test of EIS, potentiodynamic polarization curves were recorded from -1.8 V to 0 V at a scanning rate of 1.0 mV·s^−1^. All the electrochemical measurements were repeated three times to ensure reproducibility. In the immersion test, both untreated and treated samples were immersed in 20 mL Cl^−^ containing simulated concrete pore solutions for 24 h. After that, the samples were taken out, rinsed with water and ethanol, and naturally dried in air. Then, their surface morphologies after immersion were observed by scanning electron microscopy (SEM).

## 3. Results and Discussion

**Figure 1:**
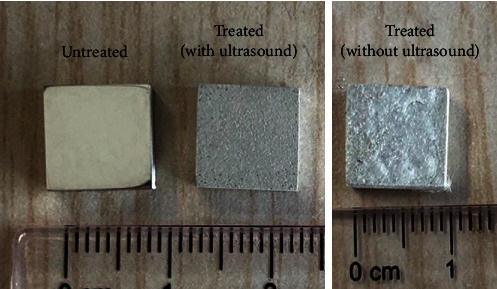
Appearance of the untreated and treated samples.

**Figure 2:**
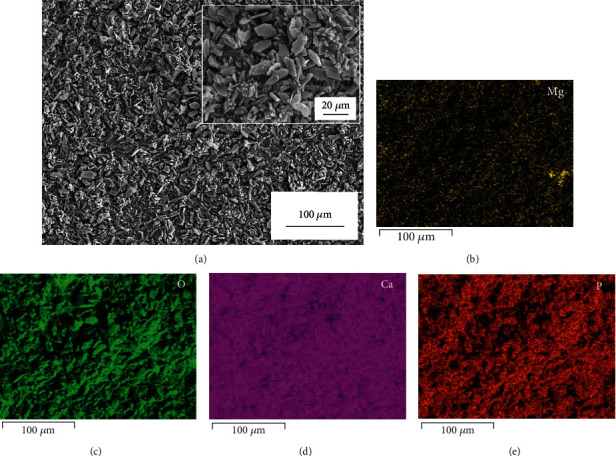
Surface morphology (a) and elemental distribution (b–e) of the treated Mg alloy. The inset in Figure 2(a) shows the surface morphology observed at higher magnification.

Figure 1 shows the appearance of the Mg-Nd alloy samples used in this study, indicating the surface of Mg alloy has been obviously changed after chemical deposition. By comparison, it is concluded that the ultrasound-assisted chemical deposition can obtain a smoother surface than that without using ultrasound. Here, it should be pointed out that the chemical deposition without ultrasound cannot meet the requirement from the view of surface quality. Therefore, we abandon the strategy of conventional chemical deposition in this study and directly choose the process of ultrasound-assisted chemical deposition.  Figure 2 exhibits the microscale surface morphologies of the treated sample observed by SEM. As shown in Figure 2(a), the treated surface is evenly covered by a layer of microflakes, and the inset acquired at high magnification shows that those microflakes pile up tightly. Figures 2(b)–2(e) further reveals the elemental distribution of elements Mg, O, Ca, and P, which means that a Ca-P coating has been formed on the surface.

**Figure 3:**
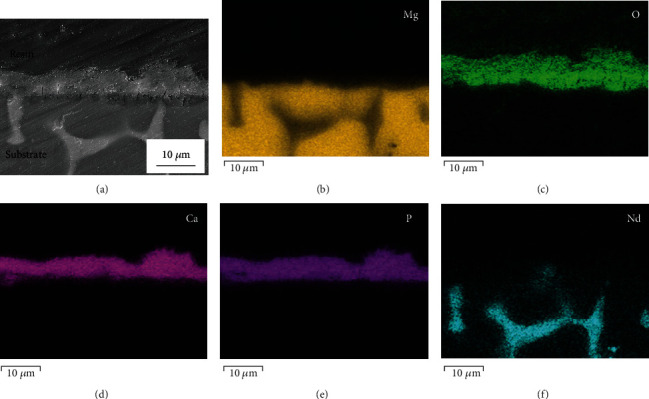
Cross-section of the treated sample: (a) SEM image and (b–f) its EDS elemental maps.

**Figure 4:**
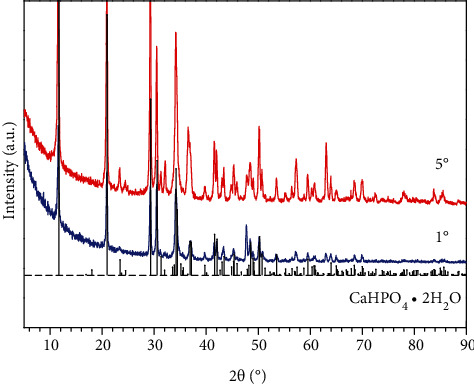
GIXRD patterns of the treated sample.

Figure 3 exhibits the cross-section of the treated sample. It can be clearly seen in Figure 3(a) that a layer of dense coating is well formed on the substrate. According to the EDS elemental maps in Figures 3(b)–3(f), the layer mainly contains Ca, P, and O. GIXRD is performed to further determine the phase composition of the coating and the corresponding results are shown in Figure 4. Due to the difference of the investigated depths, those peaks on the curve of 1° (incident angle) are weaker than those on the curve of 5° (incident angle). But the positions of those peaks are the same, which also match those of standard CaHPO_4_·2H_2_O diffraction peaks very well. Thus, it can be easily confirmed by comparison that this coating mainly consists of CaHPO_4_·2H_2_O.

**Figure 5:**
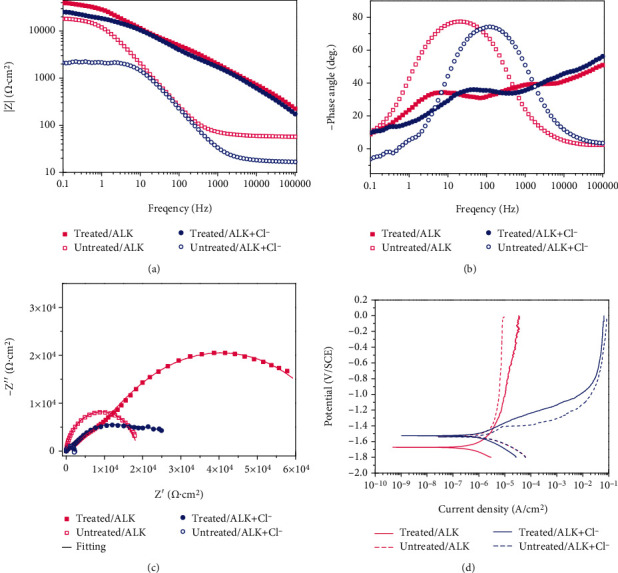
(a) Bode plots of the samples: impedance versus frequency. (b) Bode plots of the samples: phase angle versus frequency. (c) Nyquist plots of the samples. (d) Polarization curves of the samples.

**Figure 6:**
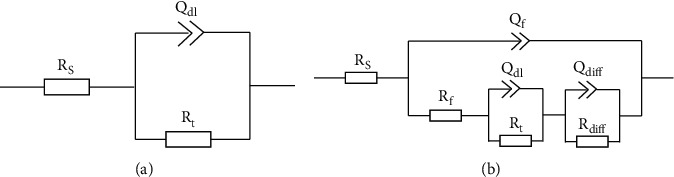
Equivalent circuit models for EIS data fitting: (a) Untreated samples [21] and (b) treated samples [22].

Figure 5 shows the results of those electrochemical tests including electrochemical impedance spectra and polarization.  Figure 5(a) gives the Bode plots of impedance versus frequency, Figure 5(b) exhibits the Bode plots of phase angle versus frequency, and Figure 5(c) draws their corresponding Nyquist plots. Here, the saturated Ca (OH)_2_ solution is denoted as ALK, and the Cl^−^ containing solution is named after ALK+Cl^−^. Both the untreated sample and treated sample have a high impedance in the saturated Ca (OH)_2_ solution, but in the saturated Ca (OH)_2_ solution diluted by 3.5 wt.% NaCl solution, the impedance of the untreated sample becomes very low. Fortunately, the treated sample changes this trend, and its impedance turns close to that of the untreated one in the Cl^−^ free saturated Ca (OH)_2_ solution, indicating that the corrosion resistance is improved by ultrasound-assisted chemical deposition. Two equivalent circuits, *R*_*s*_ (*Q*_dl_*R*_*t*_) [21] and *R*_*s*_(*Q*_*f*_(*R*_*f*_(*Q*_dl_*R*_*t*_)(*Q*_diff_*R*_diff_))) [22] in Figure 6, are proposed to fit the impedance spectra of the untreated samples and treated samples, respectively. Here, *R*_*s*_ is the solution resistance. *Q*_dl_ and *R*_*t*_ represent the double-layer capacitance and charge transfer resistance, respectively. *Q*_diff_ represents the capacitance pertaining to the diffusion, and *R*_diff_ represents the relevant resistance. *Q*_*f*_ denotes the capacitance of the deposited film, and *R*_*f*_ is the total resistance of the pores in the film. The fitted data are exhibited in Table 1. The polarization curves obtained in this study are displayed in Figure 5(d), and the corrosion potential as well as corrosion current density is derived from cathodic Tafel region extrapolation (shown in Table 2). Generally, higher corrosion current density corresponds to lower corrosion resistance. In Cl^−^ containing simulated concrete pore solutions, the corrosion current density of the treated sample has been reduced to about one-third of that of the untreated sample. Therefore, it can be found based on the data in Table 2 that the treated sample has a better corrosion resistance than the untreated one in the Cl^−^ containing simulated concrete pore solution.

**Figure 7:**
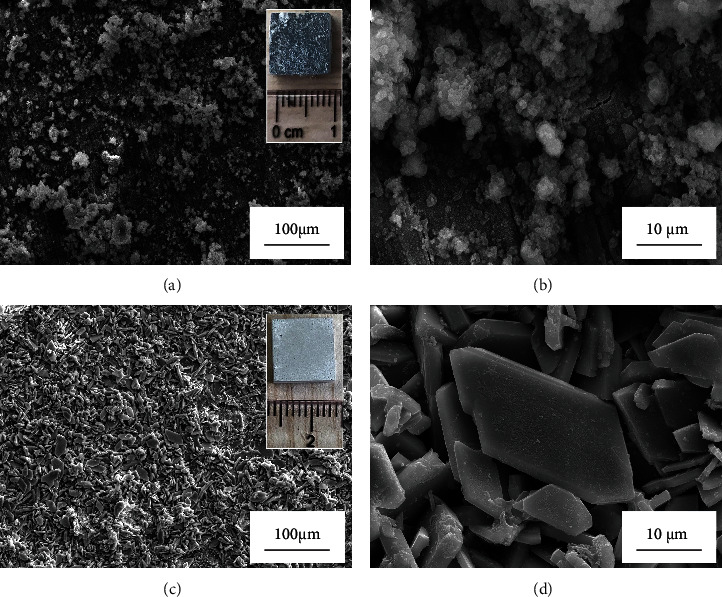
SEM images of the untreated and treated samples after immersion in Cl^−^ containing simulated concrete pore solution for 24 h: (a, b) surface morphology of the untreated sample and (c, d) surface morphology of the treated sample. The insets show the appearance of the samples after immersion for 24 h.

Figure 7 shows the surface morphology of the samples after the immersion in Cl^−^ containing simulated concrete pore solutions for 24 h. It can be seen that the untreated sample suffers from severe aqueous corrosion whereas the treated one still keeps its surface intact. Even in SEM images, it is observed that those microflakes on the surface of the treated sample are well preserved. Finally, it is confirmed that the dense coating prepared by ultrasound-assisted chemical deposition can act as an effective barrier to mitigate the corrosion of Mg-Nd alloys in Cl^−^ containing simulated concrete pore solutions.

If metallic magnesium is immersed in aqueous solutions, the overall corrosion reaction can be described as follows: Mg + 2H_2_O → Mg^2+^ + 2OH^−^ + H_2_↑ [23]. Usually, the dissolution of Mg increases the OH^−^ concentration, and magnesium hydroxide will precipitate once its solubility limit is exceeded [24]. Therefore, it can be easily understood based on the above chemical reaction that magnesium owns an immune behavior in alkaline solutions. Unfortunately, OH^−^ is prone to be replaced by Cl^−^ to form soluble chloride which expedites the dissolution of magnesium hydroxide [25]. Thus, the corrosion resistance of magnesium and its alloys will decrease significantly when Cl^−^ occurs in the simulated concrete pore solution.

A layer of dense coating is revealed in Figures 2 and 3, and here, its formation mechanism is simply discussed as follows. Firstly, H_2_PO_4_^−^ ionizes in aqueous solutions to release HPO_4_^2-^ and H^+^ [26, 27]. Next, HPO_4_^2-^ preferentially bonds with Ca^2+^ to form insoluble CaHPO_4_·2H_2_O in this acidic phosphate solution, whose related chemical reaction is shown below: Ca^2+^ + HPO_4_^2-^ + 2 H_2_O → CaHPO_4_∙2H_2_O [28, 29]. In addition, OH^−^ produced from the corrosion of magnesium will react with H_2_PO_4_^−^ to facilitate the formation of HPO_4_^2-^ [28, 29]. Finally, when the surface is uniformly covered by CaHPO_4_·2H_2_O, the corrosion resistance of magnesium alloys can be improved due to the protective effect of CaHPO_4_·2H_2_O. Nowadays, magnesium alloys have many potential applications such as formwork and reinforcing bar in building engineering due to the urgent requirement of lightweight construction. On the heels of this trend, a facile process is successfully developed in our study to enhance the corrosion resistance of magnesium alloys in Cl^−^ containing simulated concrete pore solutions.

## 4. Conclusion

A layer of dense Ca-P coating is fabricated on Mg-3.3 wt. %Nd alloy by ultrasound-assisted chemical deposition, which mainly consists of CaHPO_4_·2H_2_O. Mg-Nd alloy has a good corrosion resistance in the simulated concrete pore solution because of the immune behavior of magnesium in alkaline aqueous environments. However, it cannot resist the corrosion attack when chloride ions occur in the simulated concrete pore solution. Fortunately, this Ca-P coating can protect the substrate effectively in the Cl^−^ containing simulated concrete pore solution. In summary, it offers an economical and environmentally friendly means to produce a barrier structure on Mg-Nd alloys and further paves a potential way to develop Mg alloys as civil engineering materials in the future.

## Figures and Tables

**Table 1:** Fitted results for the different samples in simulated concrete pore solution based on the corresponding equivalent circuit models.

	Untreated/ALK	Treated/ALK	Untreated/ALK+Cl^−^	Treated/ALK+Cl^−^
Equivalent circuit model	R(QR)	R(Q(R(QR)(QR)))	R(QR)	R(Q(R(QR)(QR)))
*R*_*S*_ (*Ω* cm^2^)	59.42	225.05	17.47	101.26
*Y*_*f*_ (*Ω*^−2^ cm^−2^ s^-n^)		1.473 × 10^−7^		9.872 × 10^−7^
*n*_*f*_		0.7224		0.646
*R*_*f*_ (*Ω* cm^2^)		1864		1542
*Y*_dl_ (*Ω*^−2^ cm^−2^ s^-n^)	1.069 × 10^−5^	4.94 × 10^−6^	1.016 × 10^−5^	3.866 × 10^−6^
*n*_dl_	0.9228	0.7278	0.9392	0.8604
*R*_ct_ (*Ω* cm^2^)	1.856 × 10^4^	6.17 × 10^4^	2177	5891
*Y*_diff_ (*Ω*^−2^ cm^−2^s^-n^)		3.034 × 10^−6^		2.554 × 10^−5^
*n*_diff_		0.5772		0.3757
*R*_diff_ (*Ω* cm^2^)		9167		2.812 × 10^4^

**Table 2:** Corrosion potential and corrosion current density determined from polarization curves.

	*E*_corr_ (V/SCE)	*I*_corr_ (A∙cm^−2^)
Untreated/ALK	−1.481 ± 0.050	(3.46 ± 4.47) × 10^−7^
Treated/ALK	−1.667 ± 0.039	(3.74 ± 0.11) × 10^−7^
Untreated/ALK+Cl^−^	−1.531 ± 0.023	(6.09 ± 1.12) × 10^−6^
Treated/ALK+Cl^−^	−1.545 ± 0.019	(1.96 ± 2.51) × 10^−6^

## Data Availability

The data used in this study are available from the corresponding author upon request.
